# The Effects of Different Dynamic Culture Systems on Cell Proliferation and Osteogenic Differentiation in Human Mesenchymal Stem Cells

**DOI:** 10.3390/ijms20164024

**Published:** 2019-08-17

**Authors:** Hsiou-Hsin Tsai, Kai-Chiang Yang, Meng-Huang Wu, Jung-Chih Chen, Ching-Li Tseng

**Affiliations:** 1 Department of Dermatology, School of Medicine, College of Medicine, Taipei Medical University, Taipei City 110, Taiwan; 2 Department of Dermatology, Taipei Medical University Hospital, Taipei City 110, Taiwan; 3 School of Dental Technology, College of Oral Medicine, Taipei Medical University, Taipei City 110, Taiwan; 4 Graduate Institute of Biomedical Materials and Tissue Engineering, College of Biomedical Engineering, Taipei Medical University, Taipei City 110, Taiwan; 5 Department of Orthopedics, Taipei Medical University Hospital, Taipei City 110, Taiwan; 6 Department of Orthopedics, School of Medicine, College of Medicine, Taipei Medical University, Taipei City 110, Taiwan; 7 Institute of Biomedical Engineering, National Chiao Tung University, Hsinchu 300, Taiwan; 8 International Ph. D. Program in Biomedical Engineering, College of Biomedical Engineering, Taipei Medical University, Taipei City 110, Taiwan; 9 Research Center of Biomedical Device, College of Biomedical Engineering, Taipei Medical University, Taipei City 110, Taiwan; 10 International Ph. D. Program in Cell Therapy and Regenerative Medicine, College of Medicine, Taipei Medical University, Taipei City 110, Taiwan

**Keywords:** dynamic culture, flow reactor, mesenchymal stem cells, proliferation, differentiation, osteogenesis, bone regeneration

## Abstract

The culture environment plays an important role for stem cells’ cultivation. Static or dynamic culture preserve differential potentials to affect human mesenchymal stem cells’ (hMSCs) proliferation and differentiation. In this study, hMSCs were seeded on fiber disks and cultured in a bidirectional-flow bioreactor or spinner-flask bioreactor with a supplement of osteogenic medium. The hMSCs’ proliferation, osteogenic differentiation, and extracellular matrix deposition of mineralization were demonstrated. The results showed that the spinner flask improved cell viability at the first two weeks while the bidirectional-flow reactor increased the cell proliferation of hMSCs through the four-week culture period. Despite the flow reactor having a higher cell number, a lower lactose/glucose ratio was noted, revealing that the bidirectional-flow bioreactor provides better oxygen accessibility to the cultured cells/disk construct. The changes of calcium ions in the medium, the depositions of Ca^2+^ in the cells/disk constructs, and alkaline phosphate/osteocalcin activities showed the static culture of hMSCs caused cells to mineralize faster than the other two bioreactors but without cell proliferation. Otherwise, cells were distributed uniformly with abundant extracellular matrix productions using the flow reactor. This reveals that the static and dynamic cultivations regulated the osteogenic process differently in hMSCs. The bidirectional-flow bioreactor can be used in the mass production and cultivation of hMSCs for applications in bone regenerative medicine.

## 1. Introduction

Cell-based therapy provides a cure to repair tissue damage and several degenerative diseases. However, the production of large numbers of functional cells is still a great challenge for clinical applications [1]. It is known that terminally differentiated cells, such as chondrocytes and nucleus pulposus cells, may de-differentiate and lose their functional phenotype during in vitro cell culture expansion [2,3]. Otherwise, rapid aging, phenotype change, and stemness loss are noticed in bone marrow-derived mesenchymal stem cells (MSCs) after in vitro expansion [4]. Furthermore, the culture conditions during monolayer cell expansion are also known to affect the self-renewal, multipotency, and lineage-specific differentiation potentials of the adipose-derived stem cells [5]. Therefore, culture methods to preserve the differential potency of stem cells or to maintain a functional phenotype of terminally differentiated cells should be optimized for cell-based therapy.

It has been reported that the environmental differences between cells in a naive niche and those cultured in vitro cause phenotypic changes in stem cells [6]. On the contrary, bone marrow MSCs under dynamic culture showed a primitive stem-cell phenotype in terms of lineage-specific differentiations. A rotary bioreactor was developed to facilitate rapid expansion of human bone marrow MSCs while multiple differentiation potentials were not impaired [7]. Similarly, the rotary bioreactor was found to promote cell proliferation and maintain differentiation potentials in rat bone marrow MSCs [8]. Furthermore, it is known that the designs of bioreactors also modulated human bone marrow MSCs’ differentiations while a spinner-flask bioreactor improved osteogenic and chondrogenic differentiations when compared with the static culture and rotating-wall vessel-bioreactor culture [9]. Therefore, the bioreactor is critical for stem cell expansion and controlled differentiation during cellular manufacturing [10].

In this study, we seeded human bone marrow MSCs (hMSCs) on a non-woven fiber disk as a solid support growth matrix, and the cells/disk constructs were transferred into two different dynamic culture systems with an osteogenic medium supplement. The cell proliferation, osteogenic differentiation, and matrix deposition of hMSCs under different culture conditions were demonstrated to figure out the influence of hMSCs for in vitro cultivation.

## 2. Results

### 2.1. Cell Proliferation

**Figure 1:**
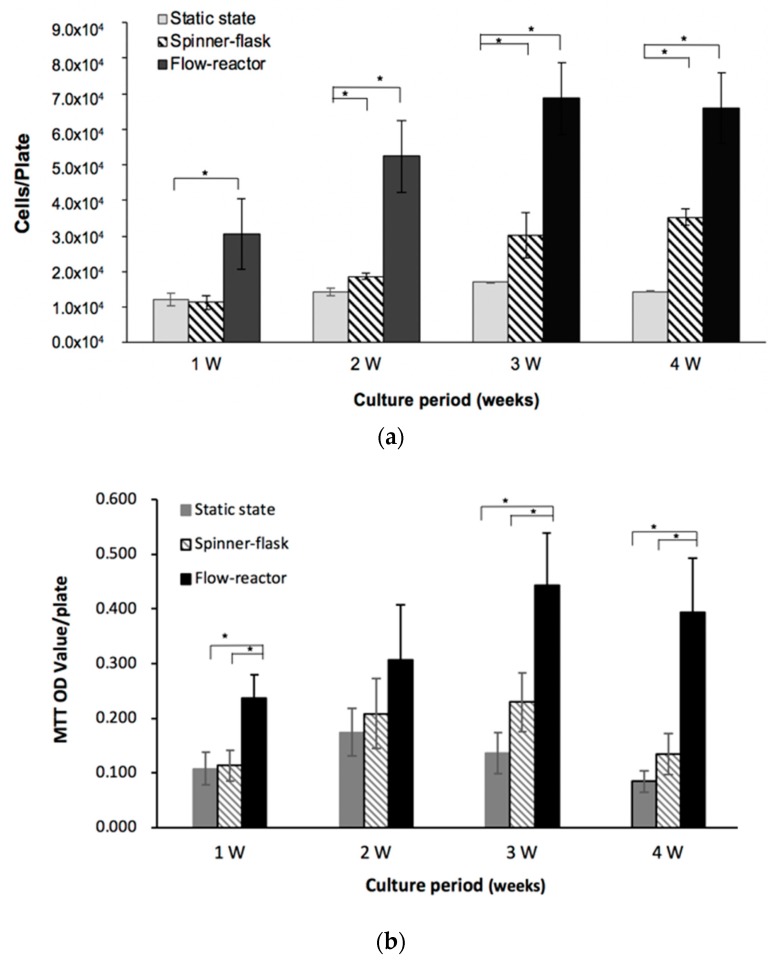
(**a**) The proliferation of human mesenchymal stem cells (hMSCs)with osteogenic medium under different culture environments was determined based on total DNA quantification, and the flow reactor had a large cell number relative to static-state culture and spinner flask; (**b**) the mitochondria activity of hMSCs under variant culture system was determined by the 3-(4,5-Dimethylthiazol-2-yl)-2,5-diphenyltetrazolium bromide (MTT) assay, and cells in the flow reactor had a relatively higher viability (*p < 0.05).

The proliferation of hMSCs with osteogenic medium under different culture environments was determined based on total DNA quantification (Figure 1a). When the flow reactor was used, a significantly higher cell number (5.2 × 10^4^) was obtained when compared with that of cells in the static-state culture (1.4 × 10^4^) or spinner flask (1.8 × 10^4^) at week one (*p* < 0.05) and week two (*p* < 0.05). However, there was no significant difference in the cell number between the static-state culture and spinner-flask bioreactor for the first two weeks. At week three, the flow reactor had a significantly higher cell number than that of the spinner-flask group (*p* < 0.05) and static-state group (*p* < 0.05), and the static-culture group also had a significantly lower cell number than that of the spinner-flask group (*p* < 0.05). Similar findings were found at week four. Besides, significant differences were found in the cell number between the spinner-flask and static-state group for the last two weeks. Overall, the cell numbers acquired from the flow-reactor culture were all much higher than the other groups during the four weeks of cultivation (Figure 1a).

The expansion rate of cells (compared with the number of initial seeding cells) further revealed that the flow-reactor had a relatively faster expansion rate than that of the spinner-flask and static-state culture at week three and week four (Table 1). Comparing with static state culture, an almost 4.6-fold larger cell number was acquired at week four in flow-reactor group, showing the efficacy for cell proliferation. Otherwise, the spinner flask just had a slightly faster expansion rate (~2.5-fold) than the static-state culture at the same time points (week 4). During the whole culture period, the DNA content in the flow reactor represented the highest cell number in all tested groups.

### 2.2. Mitochondrial Activity

The cells cultured in the flow reactor had a significantly higher cell activity than that of the static-state culture and spinner flask (both *p* < 0.05); no significant difference was noticed in the latter two groups at week one (Figure 1b). However, there was no difference in the viability among groups at week two. Otherwise, the static-state group had the lowest cell activity among groups (*p* < 0.05 for the spinner flask and flow reactor), and the flow reactor had a higher cell activity relative to the spinner flask at week three (*p* < 0.05). At week four, cells cultured in the flow reactor had the highest viability (*p* < 0.05 to the spinner-flask and the static-state group), and the static-state culture had significantly lower cell activity relative to that of the spinner-flask bioreactor (*p* < 0.05, Figure 1b).

### 2.3. Metabolite Assay

**Figure 2:**
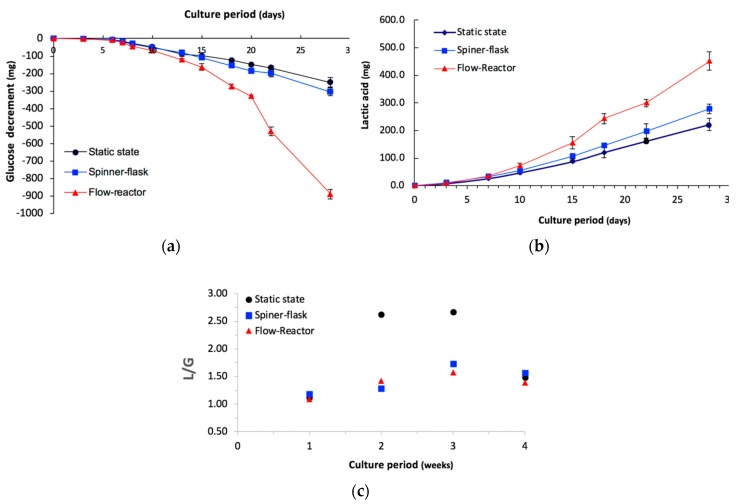
The profiles of (**a**) glucose consumption and (**b**) lactic acid production in culture media from three culture systems. (**c**) Variations in the lactic acid to glucose (Lac/Glu) mole ratio during the culture period. The static-state culture had a high L/G (Lac/Glu) ratio as represented by the anaerobic metabolism, with O_2_ shortage occurring during cultivation. However, the low Lac/Glu ratio in the flow reactor and spinner flask revealed that the dynamic culture had high amounts of oxygen and mass transportation.

The cells had relatively higher glucose (Glu) consumption (Figure 2a) and higher lactic acid (Lac) production (Figure 2b) in the flow reactor after day 14. On the contrary, cells cultured in the spinner flask had low Glu consumption and low Lac production as the metabolic profile of the static-state culture. Despite all groups having a similar Lac/Glu ratio (range 1.09–1.42) between week one to week four in the dynamic culture groups, the static-state culture had a high Lac/Glu ratio (2.67) after three weeks of cultivation (Figure 2c). However, there was no obvious difference in the Lac/Glu ratio between the flow reactor and spinner flask during the culture period. The static-state culture had a high L/G ratio, as represented by the aerobic metabolism during cultivation. On the contrary, the low Lac/Glu ratio in the flow reactor and spinner flask revealed that the dynamic culture had high amounts of oxygen and mass transportation for the culture environment.

### 2.4. Examination of Osteogenic Differentiation

**Figure 3:**
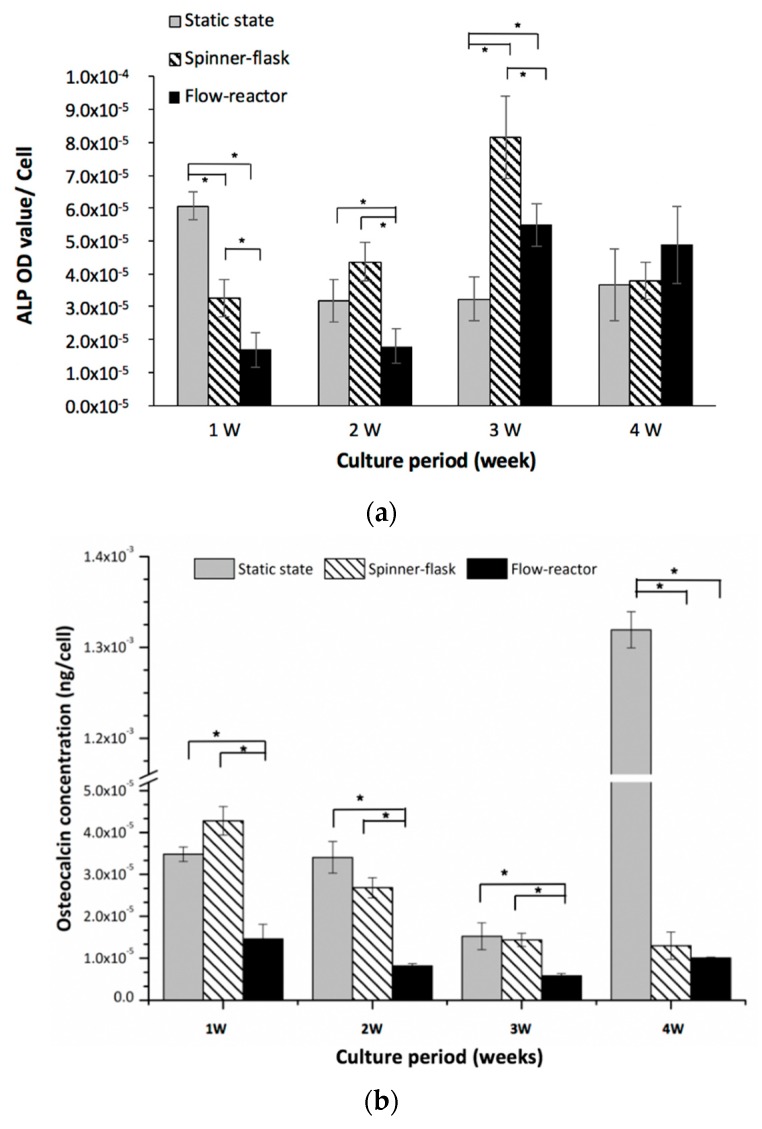
(**a**) alkaline phosphate (ALP) activity and (**b**) osteocalcin concentration in static-state, spinner-flask, and flow-reactor culture systems. The hMSCs cultured in the flow reactor had relatively lower ALP activity than those of the spinner-flask and static-state culture in the first two weeks; however, the ALP level increased dramatically at week three. Regarding the osteocalcin concentration, both the static-state and spinner-flask cultures had relatively higher levels in the first week and decreased gradually through the study while the static state had an extremely high level of osteocalcin at week four. Otherwise, the flow reactor maintained a stable osteocalcin concentration.

In the static-state culture, the highest alkaline phosphate (ALP) activity was observed in the first week, and the activity decreased thereafter (Figure 3a). On the other hand, cells cultured in the spinner flask showed the highest ALP activity at week three (*p* < 0.05 compared to the static-state culture and flow reactor). The hMSCs cultured in the flow reactor with osteogenic medium had a significantly lower ALP activity than those of the spinner-flask and static-state culture (both *p* < 0.05) in the first two weeks. After that, the ALP value increased to three times higher than the first two weeks for cells in the flow reactor (Figure 3a). No significant differences were noticed in the ALP activity among all groups at week four while the flow reactor had higher ALP activity. The osteocalcin content, a protein secreted by osteoblasts as a pro-osteoblastic factor, was examined to confirm the differentiation of hMSCs into the osteogenic linage (Figure 3b). In the static-state culture, the osteocalcin concentration in differentiated hMSCs reached 13 ng/mL/cell, which was extremely high in all groups at week four. The spinner flask had a relatively higher level in the first week and decreased gradually. Otherwise, the flow reactor had a low osteocalcin concentration but demonstrated stable secretion by the differentiated hMSCs during the culture period.

### 2.5. Medium and Intracellular Calcium Content

**Figure 4:**
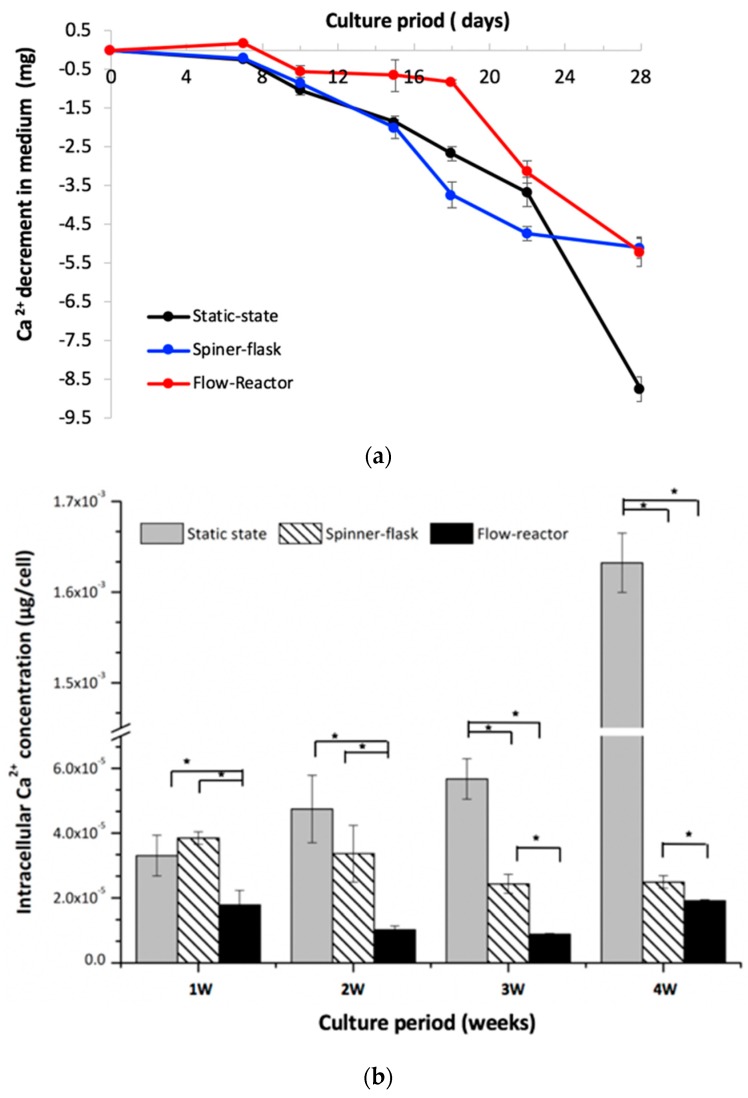
(**a**) For the Ca^2+^ concentration in culture media, there was no obvious change among the three groups in the first seven days; (**b**) for the intracellular calcium quantification, there was no significant difference between the static-state culture and spinner-flask bioreactor at week one and two. However, the static-state culture had significantly higher Ca^2+^ contents relative to the spinner flask at week three and four. The spinner flask also had significantly higher Ca^2+^ contents when compared to those of the flow reactor during the four-week experimental period.

For the Ca^2+^ concentration in culture media, there was no obvious change among the three groups in the first seven days (Figure 4a). The Ca^2+^ concentration in the media decreased gradually after day 10 for both the spinner-flask and static-state culture groups, and the static-state culture had a relatively lower Ca^2+^ concentration at day 28. Despite the flow-reactor maintaining stable Ca^2+^ concentrations before day 18, this concentration decreased dramatically after that day. For the intracellular calcium quantification, there was no significant difference between the static-state culture and spinner-flask bioreactor at week one and two. However, the static-state culture had significantly higher Ca^2+^ contents relative to the spinner flask at week three and four. Otherwise, the spinner flask also had significantly higher Ca^2+^ contents when compared to those of the flow reactor during the four-week experimental period (Figure 4b). The flow reactor had a low Ca^2+^ concentration through this study.

### 2.6. Histological Inspection

**Figure 5:**
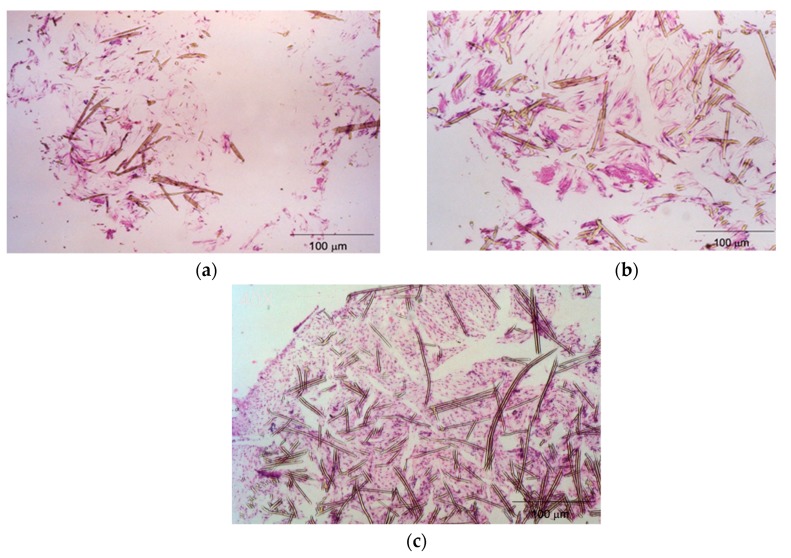
(**a**) The histological study revealed that hMSCs/fiber disk cultured under the static-state culture had a low cell number, non-uniform cell distribution, and few extracellular matrix depositions; (**b**) the spinner flask improved cell distribution while the cell proliferation and matrix deposition were unchanged; (**c**) in addition to a uniform cell distribution, the flow reactor improved cell proliferation and extracellular matrix production dramatically (scale bar, 100 μm).

The histological study revealed that hMSCs/fiber disk cultured under the static-state culture had a low cell number, non-uniform cell distribution, and few extracellular matrix depositions (Figure 5a). The spinner flask improved cell distribution while the cell proliferation and matrix deposition were unchanged (Figure 5b). In addition to a uniform cell distribution, the flow reactor improved the cell proliferation and extracellular matrix production dramatically (Figure 5c). The hematoxylin and eosin (H&E)staining ratio, which represents cells’ localization in scaffolds, in the photo was quantified by the software, ImageJ. The staining ratio in Figure 5a is around 26% for the static culture and in Figure 5b it is 36% for the spinner-flask group. Additionally, the staining ratio in the flow reactor reached 63%, which revealed that more cells exist in the 3D construct.

## 3. Discussion

Bioreactors, which can facilitate clinical-scale cell expansion and preserve differential potentials, are critical to cellular manufacturing [11]. It is known that the formation and differentiation of the human embryoid body can be improved by using a bioreactor [12]. Otherwise, bioreactors can also be used for controlled differentiation since previous studies reported that specific-lineage inductions, such as osteogenic and chondrogenic differentiations, were improved [9,13]. Enhancement of the transportation of oxygen, nutrients, and waste is another advantage of bioreactors while the exchange of these components rely on passive diffusion in static cultivations. Furthermore, the supply of oxygen and soluble nutrients is a major concern to the in vitro culture of 3D tissue-engineered constructs. Bioreactors can overcome external mass-transfer limitations and improve hypoxia-induced central necrosis in cultured tissue constructs [14].

Despite bioreactors’ ability to improve mass diffusion and avoid hypoxia, the extensional flow can result in shear stress, causing cell damage [15]. Some types of bioreactors may also generate air bubbles during operation, and bubble bursting has been shown to cause injury to cells [16]. Similarly, turbulent hydrodynamics in the bubble–liquid interphase were found to cause cell damage in a sparged bioreactor [17]. Therefore, minimizing hydrodynamic stress during cell culture is an important issue for bioreactor design [18]. The bidirectional-flow bioreactor used in this study possesses the characteristics of low shear stress and bubble-free air, thus avoiding the shortcomings mentioned above. The stirred rate for the spinner flask with cells grown on microcarriers could influence the agitation of the medium. Lock et al. [19] showed that hMSCs propagated on microcarriers in a spinner flask were sensitive to agitation speeds. High agitation speeds (80 rpm) resulted in a lower cell yield and increased the doubling time when compared to low agitation speeds (45 rpm) [20]. MSCs require slower speeds (≤20 rpm) during the initial seeding phase to promote cell attachment to microcarriers. Then, it is recommended that the agitation rate is increased to 30 rpm on the following day for culture [21]. Therefore, in this study, the stirred rate for the spinner-flask culture was 30 rpm for MSCs cultivation.

The dynamic culture of bioreactors has also been shown to have an impact on cell proliferation. Osiecki et al. found that enhancing oxygen availability in a packed-bed bioreactor improved cell proliferation to MSCs [22]. Goh et al. reported that a stirred microcarrier-culture improved the cell expansion capacity and exhibited a high osteogenic potential for human fetal MSCs [23]. Similarly, this study showed that hMSCs had a higher expansion rate than the static culture, even when the osteogenic medium was provided in these two dynamic culture systems (Figure 1, Table 1). In addition to the cell number, cells cultured in the flow reactor also had relatively higher viability during the experimental periods, especially at week three and four. A previous study reported that hypoxia can inhibit cell senescence, enhance cell expansion, maintain stemness, and even increase the differentiation potential of MSCs [24]. The total culture period lasted for four weeks in this study, and we repeated each experiment for at least two batches to verify the impact of the bidirectional-flow culture system on MSCs’ cultivation and differentiation into osteolineage cells.

Under aerobic metabolism, Glu can be effectively transferred as adenosine triphosphate through the tricarboxylic acid cycle while anaerobic metabolism produces lactic acid (Lac) simultaneously by glycolysis [25]. Therefore, the ratio of Lac production to glucose consumption can be used to identify aerobic or anaerobic metabolism during cultivation. The high Lac/Glu ratio in the static-state culture reveals that an insufficient oxygen exchange can cause an aerobic status during cultivation. On the contrary, the Lac/Glu ratio was less than 2 in both the flow reactor and spinner flask, which provides solid evidence that the dynamic culture has high oxygen transfer. Stiehler et al. reported that 3D flow culture induced proliferation and differentiation of hMSCs in the scaffold [13]. They used the radial-flow bioreactor (RFB), which can let medium flow from the periphery to the center of the scaffolds under low shear stress, allowing greater efficiency of delivery of nutrients and gas exchange, as well as the elimination of metabolic waste products [26]. The authors also claimed that RFB cultivation generated a lower shear flow condition than spinner flaks [26]. A similar result was also found in our flow-reactor; with good nutrients/gas/waste exchange, hMSCs were maintained in an aerobatic environment (Figure 2). Furthermore, despite the bidirectional-flow bioreactor having a large cell number, these cells still showed a low Lac/Glu ratio relative to those in the spinner-flask bioreactor, which indicates that the bidirectional-flow bioreactor had a better oxygen/mass transportation capacity than the spinner flask.

During the process of bone formation, the activation of ALP is critical for the bone matrix maturation and subsequently the calcium deposited for mineralization [27]. The highest ALP activity was found at week one by the static culture. Relative to the static-state culture, the hMSCs cultured in the spinner flask showed high ALP activity at week two and three, while cells in the flow reactor had high ALP activity at week three and four (Figure 3), thus representing a different osteo-differentiated timetable. Compared with the results of Nishimura’s study, in the static culture, the cell number slightly increased from 7 to 14 days of cultivation, and ALP activity was also high on day 7 and then decreased thereafter. Runt-related transcription factor 2 (Runx2) protein is known to be detected first in pre-osteoblasts, and its expression is upregulated in immature osteoblasts and downregulated in mature osteoblasts [28] Although, we did not analyze the synthesis of Runx2 in hMSCs, alternatively, we detected the expressions of ALP and osteocalcin to identify bone cells, which also verifies the process of osteogenic differentiation to MSCs.

About bone mineralization, the calcium concentration of the osteogenic medium remained unchanged for the first week in all groups. After that, the hMSCs cultured under the static-state and spinner-flask bioreactor decreased calcium ions continually in a linearly fashion till the end of this study (Figure 4). In the flow reactor, the calcium concentration in medium remained unchanged until day 18, after which the calcium concentration decreased dramatically. Meanwhile, the cells in the flow reactor had a low calcium content, while the static-state culture had a high calcium concentration at week four (Figure 4). The calcium deposition in the extracellular matrix proceeding through Ca^2+^ acquisition from the medium represents the mineralization progression of hMSCs. Stiehler et al. observed a significantly increased calcium content in dynamically cultured cell/scaffold constructs compared with statically cultured constructs [13]. Dynamic cultivation of hMSCs by RFB revealed this dynamic culture could facilitate osteogenic differentiation due to shear stress caused by medium perfusion and enhanced nutrition delivery of medium [26,29]. In our study, the same trend was also found for the static and dynamic culture results. Effective circulation of the medium provides the necessary differentiation components to hMSCs in scaffolds in a uniform manner.

Although it is also a dynamic culture, a variant dynamic culture system with a different flow rate or shear force generation could result in a change of the timetable for MSCs’ proliferation and differentiation [13]. The benefits of flow shear stress on the proliferation of hMSCs may depend on the flow rate and the type of bioreactor, cell, scaffold, or medium [26,30]. Not all dynamic flow systems can obtain the same osteo-simulated outcome. In Birru et al.’s study [31], a new perfusion-based bioreactor was developed. This study reported that dynamic fluid flow culture enhanced ALP, Ca^2+^, and collagen activities to differentiated cells. In addition, the expression of the osteogenic markers, Runx2 and osteonectin, were also increased in cells. Despite the differences in osteonectin production, similar findings were noticed in cell expansion, cell activity, and ALP as our study. Compared to our results, a major difference is that they used umbilical cord blood MSCs. Generally, umbilical cord blood MSCs are known to have better stemness than bone marrow MSCs. However, the cell source of umbilical cord blood MSCs was not provided. In addition, shear stress, which is generated by perfusion bioreactors, may not only enhance the osteogenic differentiation of MSCs but also cause cell damage. On the contrary, the concerns of extensional flow, the generation of air bubbles, and turbulent hydrodynamics are eliminated in our bidirectional-flow culture system. Otherwise, their study showed that the compositions of the cell carrier polylactic acid/polyethylene glycol (PLA/PEG) also influenced cell proliferation as well as differentiation. Furthermore, they reported that a dynamic cell culture did not result in cell senescence in terms of the activity of senescence-associated β-galactosidase (β-gal) [31]. All these findings reveal that the osteogenic process can be modulated by the dynamic culture.

Histological inspections revealed that hMSCs cultured in the flow reactor had a large cell number and uniform cell distribution (Figure 5). The bidirectional flow condition induced mixing of oxygen and nutrients throughout the flow medium and reduced the concentration boundary layer at the construct [32], which may be attributed to this finding. Moreover, abundant extracellular matrixes were noticed, which revealed that the bidirectional flow bioreactor also regulated extracellular matrix production.

## 4. Materials and Methods

### 4.1. Cultivation of Human Bone Marrow MSCs in Bioreactors

**Figure 6:**
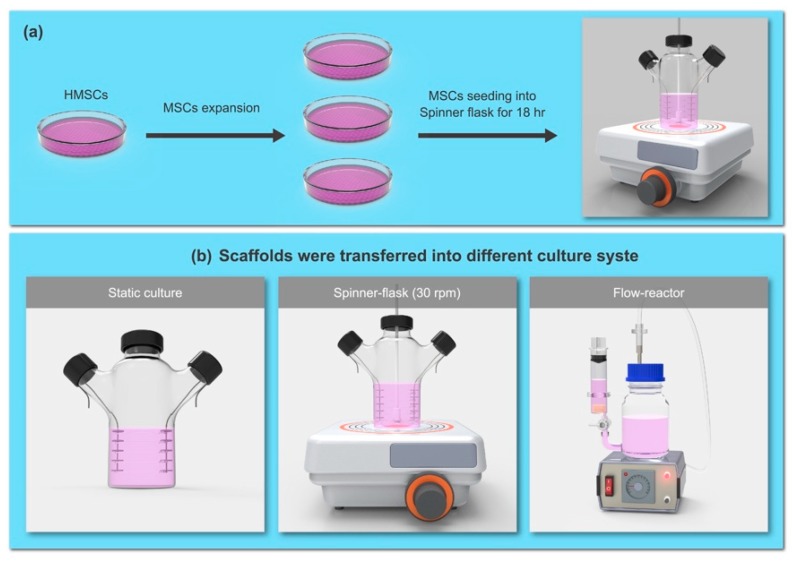

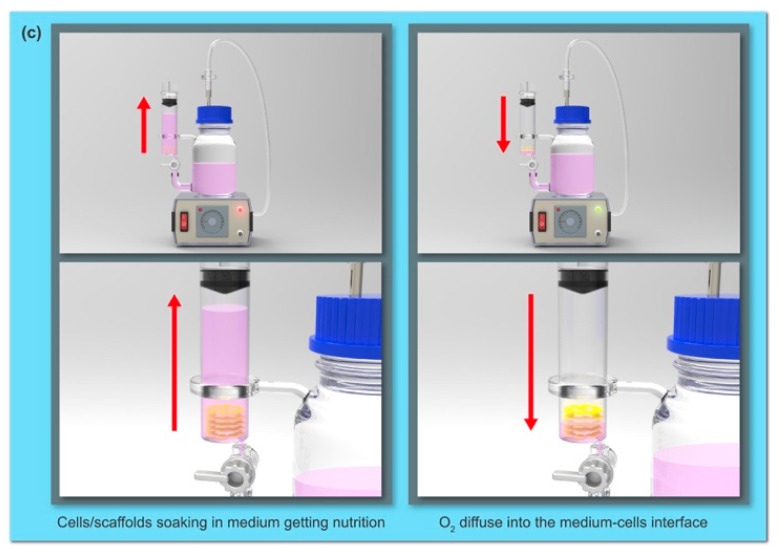
Schematic depiction of hMSCs’ (**a**) expansion in dishes and seeded onto a non-woven fiber disk; (**b**) these hMSCs/disk constructs were subsequently transferred into either a spinner-flask or a bidirectional-flow bioreactor; cells cultured in the non-stirred flask were used as a static culture. The (**c**) shows the medium flow in the culture chamber (left) and medium flow out of the culture chamber (right) in the bidirectional-flow reactor.

Human bone marrow MSCs were purchased from PromoCell (P2, C-12974, Heidelberg, Germany) and routinely cultured in its basal medium, MSC growth medium 2, C-28009, PromoCell) supplemented with 1% antibiotic solution (P4083, Sigma-Aldrich, Saint Louis, MO, USA) at 37 °C in 5% CO_2_. The culture medium was changed every 2 to 3 days, and cells at 70% to 80% confluence were detached and sub-cultured. During the expiation from P2-P5, the medium was gradually changed into αMEM plus 10% FBS (Hyclone, Logan, UT, USA) and antibiotics in our lab. The αMEM contains glucose at a concentration of 1g/mL. After initial expansion (from P2–P5) in the culture dish as a monolayer (Figure 6a), the hMSCs (P6) were seeded onto a non-woven fiber disk (polyester mesh with polypropylene support, Fibra-Cel^®^ Disk, Eppendorf, Hamburg Germany) at the cell density of 3 × 10^5^/cm^3^ disk volume. The cell/disk constructs were placed on a spinner flask overnight to allow for cell adhesion on the scaffolds, and subsequently transferred into either a spinner flask (with a 30 rpm stirred rate) or a bidirectional-flow bioreactor [32], and modifications were used (flow reactor, Figure 6b). After cultivation in the αMEM plus 10% FBS medium for 24 h, the osteogenic medium containing 10 mM Na-β-glycerophosphate, 50 mg/mL ascorbic acid, and 10^−8^ M dexamethasone was provided to the cell/disk constructs and cultured for 4 weeks. For comparison, the cell/disk constructs were also cultured in a spinner flask under quiescent conditions as a static cultivation (static state) group.

### 4.2. Total DNA Quantification for Cell Proliferation

Total DNA of cultured hMSCs was extracted from the samples at pre-determined intervals to determine cell proliferation (every week, for a total of 4 weeks). The cell/disk constructs (*n* = 6 in each group at each time point) were digested in a papain solution (P4762, Sigma-Aldrich) at 60 °C for 16 h. Total DNA of the digested samples was extracted (DNeasy Blood and Tissue kit, 69504, QIAGEN, Hilden, Germany), and the DNA content was quantified using a spectrophotometer (Infinite 200 Pro, Tecan, Switzerland). The actual cell number was determined using a standard curve established from hMSCs at different cell densities. The expansion rate of hMSCs under different culture conditions was determined. The statistic analyzed was performed by using Excel for one-way ANOVA.

### 4.3. Mitochondrial Activity

The cell activity of hMSCs in the disks under static and dynamic cultures was determined using a colorimetric 3-(4,5-Dimethylthiazol-2-yl)-2,5-diphenyltetrazolium bromide (MTT) assay (M2128, Sigma-Aldrich) every week. After being cultured in osteogenic medium, the hMSCs/disks were washed twice with PBS and subsequently cultured in medium containing 0.5 mg/mL MTT reagent for an additional 4 h to evaluate the mitochondrial activity of cells. Finally, the insoluble formazan crystals were dissolved in dimethyl sulfoxide, and the results of the MTT assay were determined using a spectrophotometer at the wavelength of 570 nm.

### 4.4. Metabolite Assay

The culture media were collected from the bioreactors and static-state group twice a week, and the concentrations of glucose (Glu) and lactic acid (Lac) in the media were determined using the BioProfile^®^ Automated Chemistry Analyzers (Nova Biomedical Corporation, Waltham, MA, USA) [32]. In addition, the ratio of lactic acid production to glucose consumption (Lac/Glu) was calculated.

### 4.5. Examination of Osteogenic Differentiation

The activity of alkaline phosphatase (ALP) or osteocalcin was determined for the differentiated cells. After being cultured in osteogenic medium, the cells on the non-woven fiber disk were lysed for protein extraction and reacted with the solution containing Tris-HCl buffer (pH 9.5), 15 mM *p*-nitrophenyl phosphate, and 0.1% Triton X-100. The reaction was stopped using 0.1 N NaOH solution, and the results were measured using a spectrophotometer (Multiskan GO Microplate Spectrophotometer; Thermo Fisher Scientific) at a wavelength of 405 nm for ALP evaluation.

The osteocalcin quantification was examined by a Gla-type osteocalcin (Gla-OC) EIA Kit (MK111, TaKaRa, Japan) following the manufacturer’s instructions, and the results of the optical density (OD) value were determined at 450 nm using a microplate reader. The calcium concentration in the culture medium was estimated using a calcium assay kit (701220, Cayman, MI, USA), and the result of the OD value was read at 575 nm with a spectrophotometer. In addition, the calcium content (extracellular and intracellular calcium ions) was also determined to confirm the calcium deposition in the hMSCs for mineralization.

### 4.6. Histological Inspection

After 4 weeks of vitro culture in the bioreactors, these cells/disk samples were harvested and fixed in 10% neutral buffered formalin for 3 days, decalcified in 10% ethylenediaminetetraacetic acid (EDTA) solution (pH 7.4), and prepared for histology examination. The paraffin blocks were cut into 5-μm slides in consecutive sections, deparaffinized using non-xylene, and stained with hematoxylin and eosin (H&E, 3008-1&3204-2, Muto, Japan).

### 4.7. Statistical Analysis

Data are expressed as mean ± standard deviation. ANOVA was used to determine the differences among groups, and differences were considered significant when the *p*-value was less than 0.05.

## 5. Conclusions

The design of dynamic culture systems has impacts on hMSCs’ proliferation and differentiation. During the four-week culture period with osteogenic medium, the bidirectional-flow bioreactor improved the cell viability and increased cell proliferation of hMSCs through the experimental period. Despite the flow-reactor having a relatively higher cell number, a relatively lower Lac/Glu ratio was noted, showing cells’ growth in aerobic conditions. This finding reveals that the flow reactor provides better mass/oxygen transportation to the cultured cells/disk constructs compared to the conventional spinner-flask and static culture. The changes of calcium ions in the medium, the depositions of calcium ions in the cells/disk construct, and the ALP activity showed these two dynamic culture systems (spinner flask vs. flow reactor) regulated the osteogenic process differently. Moreover, cells were distributed uniformly with abundant extracellular matrix production when the flow reactor was used. This study confirmed that the bidirectional-flow bioreactor is the best culture system. hMSCs can be proliferated in this culture condition and can also be differentiated into osteolineage thereafter. This fulfills the clinical point of getting more cell numbers for transplantation, while also having the capacity to accommodate the surrounding bone environment.

## Figures and Tables

**Table 1:** The hMSCs’ expansion rate in different culture systems.

Period	Static State	Spinner Flask	Flow Reactor
1 week	1.08 ± 0.17	1.00 ± 0.19	2.70 ± 0.04 ^*,#^
2 weeks	1.27 ± 0.10	1.65 ± 0.10^*^	4.64 ± 0.36 ^*,#^
3 weeks	1.51 ± 0.02	2.68 ± 0.64^*^	6.08 ± 0.21 ^*,#^
4 weeks	1.27 ± 0.02	3.13 ± 0.23^*^	5.85 ± 0.02 ^*,#^

*p < 0.05 compared with static; ^#^*p* < 0.05 compared with the spinner flask.
